# The Effectiveness of the Feldenkrais Method: A Systematic Review of the Evidence

**DOI:** 10.1155/2015/752160

**Published:** 2015-04-08

**Authors:** Susan Hillier, Anthea Worley

**Affiliations:** ^1^International Centre for Allied Health Evidence, Sansom Institute of Health Research, School of Health Science, University of South Australia, P.O. Box 2471, Adelaide, SA 5001, Australia; ^2^School of Health Science, University of South Australia, P.O. Box 2471, Adelaide, SA 5001, Australia

## Abstract

The Feldenkrais Method (FM) has broad application in populations interested in improving awareness, health, and ease of function. This review aimed to update the evidence for the benefits of FM, and for which populations. A best practice systematic review protocol was devised. Included studies were appraised using the Cochrane risk of bias approach and trial findings analysed individually and collectively where possible. Twenty RCTs were included (an additional 14 to an earlier systematic review). The population, outcome, and findings were highly heterogeneous. However, meta-analyses were able to be performed with 7 studies, finding in favour of the FM for improving balance in ageing populations (e.g., timed up and go test MD −1.14 sec, 95% CI −1.78, −0.49; and functional reach test MD 6.08 cm, 95% CI 3.41, 8.74). Single studies reported significant positive effects for reduced perceived effort and increased comfort, body image perception, and dexterity. Risk of bias was high, thus tempering some results. Considered as a body of evidence, effects seem to be generic, supporting the proposal that FM works on a learning paradigm rather than disease-based mechanisms. Further research is required; however, in the meantime, clinicians and professionals may promote the use of FM in populations interested in efficient physical performance and self-efficacy.

## 1. Introduction

The Feldenkrais Method (FM) was developed over a period of decades in the last century by Dr. Moshe Feldenkrais. He claimed the basis of the approach was founded in the human potential for* learning how to learn* [1]. As such, he operationalized an experiential process or set of processes, whereby an individual or a group could be guided through a series of movement- and sensation-based explorations. The purpose of these explorations was to practise the nonlinear process of sensing the difference between two or more options to achieve the stated movement task, and making a discernment about which may feel easier, that is to say, performed with less effort. These perceptual discernments are predicated on a judgement that is positive (pleasurable, easy, and with less effort) compared with experiencing a less favourable feedback signal such as pain, strain, or discomfort. Further to this, the participants are encouraged to generate many alternative movement solutions to the guided task to increase the opportunity for further distinctions and improvements to be made. Thus the process of intention, action, gaining feedback, making decisions, and reenacting with adaptations constitutes the learning framework in a somatic context [2].

The two modes of delivery that are offered to the public are either individual, manually directed lessons (functional integration, FI) or group, verbally directed classes (awareness through movement, ATM). The nomenclature for both reflects the fundamentals of the approach—that movement has to be based on a* functional *or meaningful intention for the system to engage and that by becoming* aware *of what and how we act (move) we become in a better place to choose an alternative behaviour (movement pattern) [3]. Both modes of delivery apply the same principles of perceptual exploration through movement that is passively and/or actively performed.

The method has been applied in varied domains across countries, from general education or children with learning issues to enhancing performance in sports and theatre. The clinical applications have received the most interest in the published literature because of the intuitive appeal of basing a health recovery process on a learning paradigm and because of the inherent fostering of self-efficacy that occurs particularly in a group setting.

In the climate of evidence-based practice in the health domain, any approach being offered to the public is being scrutinized for evidence of effectiveness and, if effective, for what type of benefit and of what magnitude for any clinical population. An earlier systematic review of the evidence for the method was published in 2005 by Ernst and Canter [4]. This review included six randomised controlled trials (RCTs) of low to moderate quality in populations such as people with multiple sclerosis, chronic low back pain, and neck issues. They concluded that there was promising evidence but its credibility was tempered due to the low number of studies, high level of clinical heterogeneity between studies, and methodological flaws. The methods employed by Ernst and Canter [4] were robust for the time; however, their risk of bias assessment used a now discarded tool (the Jadad) and their search covered until 2003. Therefore, it is timely to systematically update the evidence for the Feldenkrais Method with current review procedures.

This review had the aims of

1. systematically identifying and appraising the evidence for the effectiveness of the Feldenkrais Method across domains;
2. determining what is the nature and order of magnitude of any beneficial effects and for which population/s.

## 2. Materials and Methods

### 2.1. Criteria for Considering Studies for This Review

We employed systematic review methods based on the PRISMA guidelines [5].

### 2.2. Types of Studies

We considered all types of primary studies in the first instance in order to fully explore the potential populations and outcomes covered. In the final inclusion only studies with a random allocation and a stated control group were included. Any secondary researches (systematic and semisystematic reviews) found were not included, but rather their included studies were retrieved in full and added to the potential pool in order for all primary studies to be appraised with a consistent method.

### 2.3. Types of Participants and Outcomes

We included any population where there was an outcome of interest related to improvement in health and/or function.

### 2.4. Types of Interventions and Comparisons

Either form of Feldenkrais Method (functional integration or awareness through movement) was included as the sole approach for the intervention group. The comparison group could include placebo, inactive control, or an alternate method.

### 2.5. Search Methods for Identification of Studies

We searched the databases of AMED (Allied and Complementary Medicine), Embase Classic + Embase, Ovid MEDLINE(R), Cochrane, PsycINFO, PubMed, and Google Scholar from inception to July 2014. We considered all languages (the search was open to all listed journals irrespective of language) and publication status (we would include unpublished trials wherever found, e.g., through experts in the field or grey literature such as organizational websites).

The search terms included variations and combinations of methodology terms (such as randomised, trial, clinical, and controlled), with intervention terms such as Feldenkrais Method, (awareness through movement and functional integration). An example of the terms employed in the electronic search strategy is presented in Table 1.

From the generated lists from each database, duplicates were removed and the first high level sift was performed by one author based on title alone. The second level of review was performed by both authors and required retrieval of the abstract at the minimum. The retained studies were examined in full to confirm inclusion. Those excluded were recorded with reasons.

All retrieved studies were checked for additional references, and experts in the field were contacted to assist in identifying any further studies published or unpublished. Experts were provided from the membership of peak FM bodies (the Australian Feldenkrais Guild and the International Feldenkrais Federation) and were asked to supply further papers by email.

### 2.6. Data Collection and Analysis

Relevant data were extracted from each of the included studies using a standard trial summary sheet by one author and checked by the second. Data included author, date, study design, population sample, intervention, comparison, outcome measures, results, and comments. A risk of bias evaluation was also performed for each study by one author using standard Cochrane tables [26] with checking and data entry by the second author. Any disagreements were resolved by consensus, with a third party if necessary.

Where possible, data were extracted for meta-analyses. We planned to extract and analyse data to calculate individual and total effect sizes through odds ratios or mean differences (fixed effect or random effect if the studies were small and/or heterogeneous) and 95% confidence intervals. Statistical heterogeneity would be evaluated based on visual inspection of forest plots and on the *I* ^2^ statistic. It was not anticipated that any other analyses would be possible (e.g., subgroup or publication bias) due to a paucity of studies.

If we found that meta-analyses were not possible, then results would be synthesized and reported narratively.

## 3. Results

### 3.1. Included Studies

**Figure 1:**
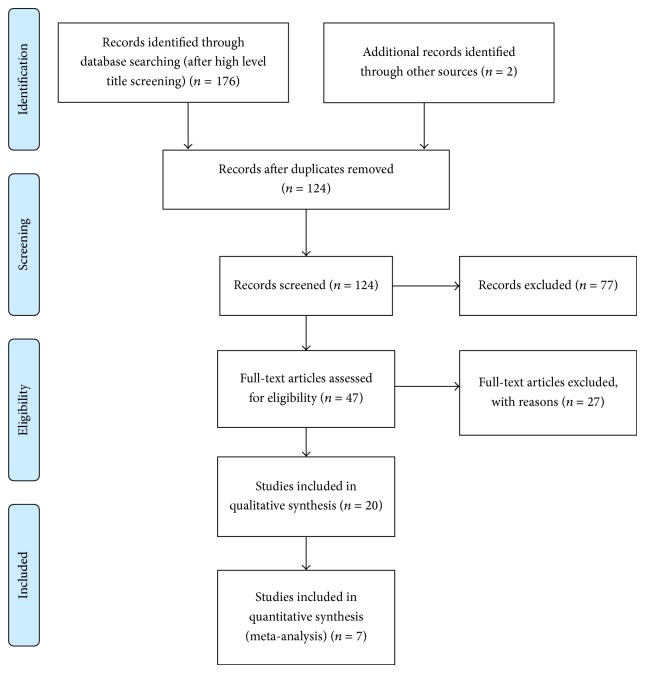
PRISMA flow diagram.

The systematic search yielded over 1,300 initial titles for high pass screening. See Figure 1 for the PRISMA Flow diagram. With duplicates and obviously irrelevant titles removed, 124 records were considered at the abstract level by both authors, with an additional two studies provided from experts in the field (newly published, one RCT, one non-RCT). Seventy-seven abstracts were excluded at this stage because they were did not report an investigation of the FM and/or did not involve a trial of effect. Forty-seven full-text articles were reviewed against the criteria and further 27 excluded with reasons noted in Table 2.

Fourteen new RCTs were included along with the original six studies from the Ernst and Canter [4] review. See Table 3 for details of all included studies. From this total of 20 studies, there were seven studies sufficiently homogenous to allow for meta-analyses.

### 3.2. Description of Studies

Publication dates ranged from 1991 [12] to 2014 [25]. Populations under investigation in the included RCTs ranged from healthy volunteers [6, 12, 15–17, 19, 24], healthy ageing [21–23], institutional ageing [25], people with multiple sclerosis [7–11, 13], eating disorders [14], myocardial infarct [18], and sleep bruxism [20]. Studies generally had small sample sizes with a mean of 40.8 participants (SD 23.5).

The nature of the Feldenkrais interventions also varied in delivery mode, intensity, and frequency. The predominant methods were single or multiple ATM lessons delivered either in a group or individually using audio recording. The comparison groups were most commonly an alternate form of therapy. Fourteen trials had active controls (such as relaxation classes or generic movement/balance classes) and six had a passive or inactive control (usual activities/no intervention).

Outcomes were also highly heterogeneous in keeping with the needs of the diverse populations and are listed in Table 3. The measures related to performance or activity outcomes (e.g., balance or dexterity), symptoms (e.g., pain, effort or mood) or were related to quality of life.

### 3.3. Excluded Studies

Table 2 summarises the list of studies (27) that were retrieved but excluded. Reasons for exclusion were predominantly around design: two were systematic reviews; five were controlled trials (not randomly allocated); eight had no control group; eight were nonsystematic reviews; one was not exclusively Feldenkrais in the intervention group; one was a content analysis of an intervention; one was a phenomenological analysis; and one was a commentary.

### 3.4. Risk of Bias in Included Studies

**Figure 2:**
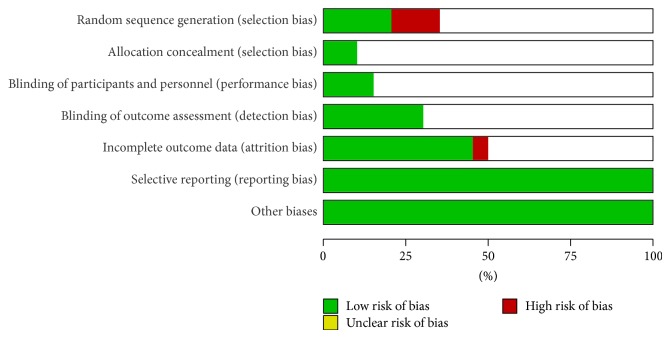
Risk of bias graph: review authors' judgements about each risk of bias item presented as percentages across all included studies.

**Figure 3:**
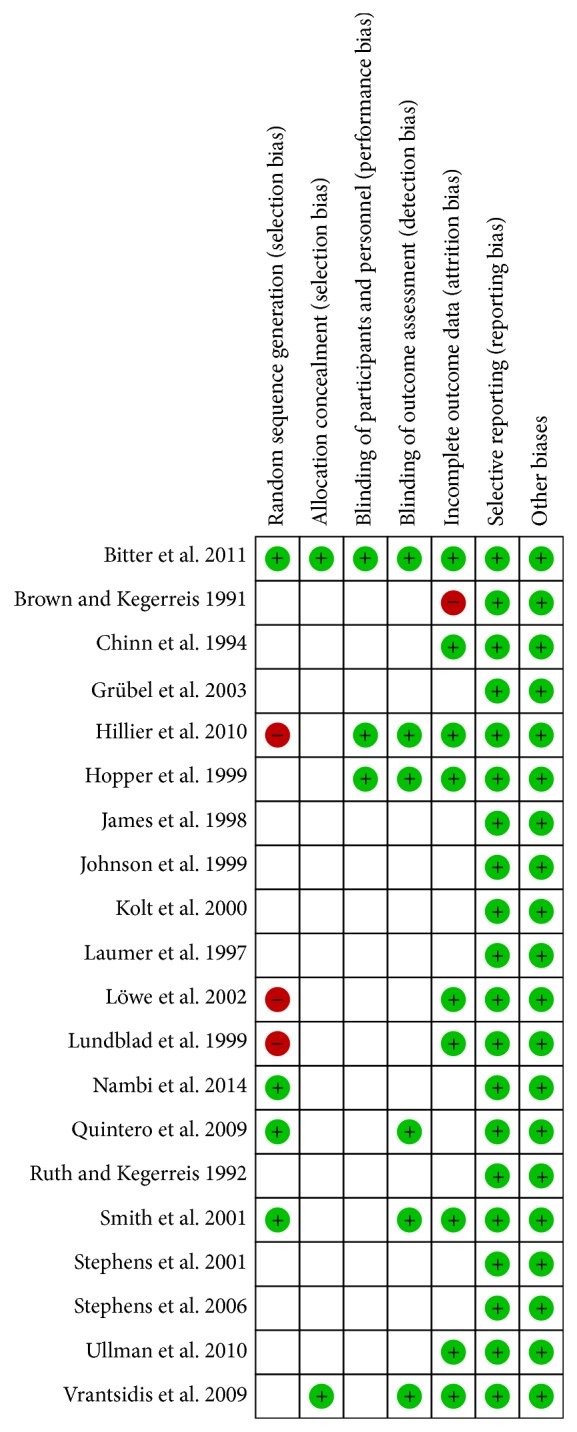
Risk of bias summary: review authors' judgements about each risk of bias item for each included study.

Risk of bias was high in most studies. Less than a quarter of the studies had adequate random allocation processes and only a third had blinding of outcome assessments. It has to be acknowledged that for trials requiring an intervention like Feldenkrais it may be difficult or inappropriate to expect blinding of therapists or even participants, though participants can be blinded to the intervention of interest if there is a plausible comparison group (such as a relaxation or other forms of movement-based class). Figures 2 and 3 summarize the risk of bias analysis. It can be seen that a definitive judgement could not be made in many cases as it could not be confirmed whether there was a clear risk of bias (given a red status) or whether the authors had simply not stated the process in sufficient detail for a judgement to be made; hence the risk of bias indicator was left blank.

### 3.5. Effects of Interventions

Sufficiently homogenous data (same population, intervention, comparator, and outcome measure) were able to be extracted to perform meta-analyses in the areas of balance training in ageing populations.

**Figure 4:**
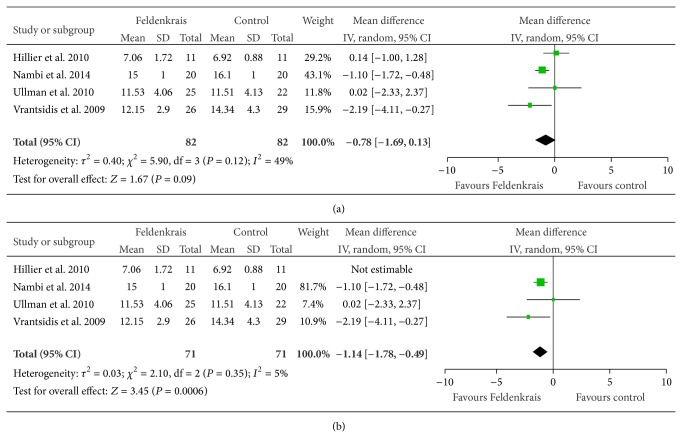
(a) Effect sizes of Feldenkrais versus control for the timed up and go test (measured in seconds; balance and mobility). (b) Effect sizes of Feldenkrais versus control for the timed up and go test (measured in seconds; balance and mobility) with Hillier 2010 removed (control group was alternate balance class).

Four studies [21–23, 25] reported on the timed up and go assessment for balance and mobility, just failing to find in favour of Feldenkrais classes (Figure 4(a)); pooling postintervention measures gave a mean difference of −0.78 s (95% CI −1.69, 0.13), *P* = 0.09. However, heterogeneity was high (*I* ^2^ = 49%). Therefore, a sensitivity analysis was performed as one study by Hillier et al. [23] compared Feldenkrais to another balance class whereas the other three studies compared the FM class to wait list control or no class. Removal of Hillier et al. [23] (Figure 4(b)) revealed a larger effect size with a mean difference of −1.13 (95% CI −1.7, −0.56), *P* = 0.0001, and heterogeneity reduced to a negligible level (*I* ^2^ = 5%). It was also noted that Nambi et al. [25] had narrow outcome variability which led to a heavier weighting in the meta-analysis.

**Figure 5:**
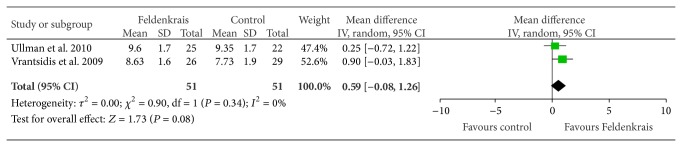
Effect sizes of Feldenkrais versus control for the Falls Efficacy Scale (balance confidence).

Two studies [21, 22] evaluated balance confidence using the Falls Efficacy Scale after FM classes (Figure 5). Pooled results trended in favour of the FM, however, failed to reach significance (MD 0.59, 95% CI −0.08, 1.26; *P* = 0.08).

**Figure 6:**
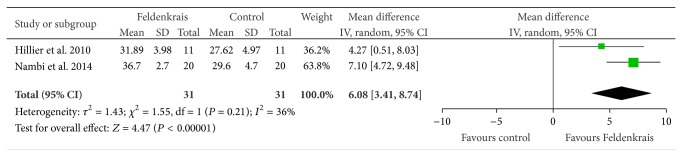
Effect sizes of Feldenkrais versus control for the functional reach test (measured in cm; balance).

Two studies [23, 25] evaluated balance using the functional reach test after FM classes (Figure 6)—pooled results found in favour of the FM classes (compared to nothing or another generic balance class) with a mean difference of 6.08 cm (95% CI 3.41,8.74), *P* < 0.00001.

**Figure 7:**
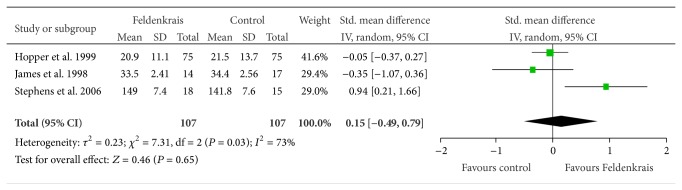
Effect sizes of the Feldenkrais Method on the active knee extension test.

Meta-analysis was also able to be performed using three studies measuring the influence of FM classes on hamstring length in healthy populations [15, 16, 19]. The authors all reported the measure as an active knee extension test; however, on visual inspection, the results appeared heterogeneous in terms of magnitude and range; therefore, a standardized mean difference (rather than MD) was calculated. No significant effect was found after the intervention compared to control (SMD 0.15, 95% CI −0.49, 0.79; *P* = 0.65) and statistical heterogeneity was unacceptably high (*I* ^2^ = 73%) (Figure 7).

Single randomised controlled studies reported statistically significant, positive benefits compared to control interventions and included the following:

- greater neck flexion and less perceived effort after a single FM lesson for neck comfort [6]; reduced prevalence of neck pain and disability in symptomatic women after FM (individual and group sessions compared to conventional care or home exercises) [8]; reduced perceived effort in FM group for people with upper torso/limb discomfort [13];
- improved balance in people with MS after eight FM sessions [9];
- improved body image parameters in people with eating disorders after a nine-hour FM course [14];
- reduction in nocturnal bruxism in young children after 10-week course of FM lessons [20];
- improved dexterity in healthy young adults after a single session of FM class [24].

Seven of the 20 studies failed to show any superior positive effects of FM compared to other comparison modalities. See Table 3 for details. No studies reported adverse events.

## 4. Discussion

### 4.1. Summary of Main Results

The majority of the 20 included studies reported significant positive effects of FM in a variety of populations and outcomes of interest. A high risk of bias/poor methods reporting does temper the interpretation of these findings. The low amount of confirmed/reported adherence to best practice conduct of RCTs may be partially attributable to the age of the studies when knowledge in the area of trial conduct was less.

Nevertheless meta-analyses in the area of balance training in ageing populations were found in favour of the FM classes for clinical measures such as the timed up and go and functional reach tests. Both of these measures are predictive of falls risk. Whilst the TUG effect size was probably not clinically significant (1- to 2-second change), the functional reach test effect size would arguably indicate a clinically meaningful change (able to reach further 6 cm).

Given the positive effects in particular outcome domains it is interesting to speculate on the mechanism of action of the FM; however, it is to be noted that this was not the purpose of the review. The favourable evidence for reduced perceptions of effort, improved dexterity, improved comfort and reduced bruxism all support the proposed mechanism of action via promotion of awareness, relaxation and more efficient action. Inconsistent results were found for improving hamstrings length indicating that a “relaxation” effect may be variable.

The populations varied in age and diagnosis indicating that a beneficial effect is possible across different domains; again this is consistent with the use of the FM in diverse populations and also consistent with the notion that it is not a healing or disease-specific mechanism of action but rather one based on more generic learning and self-improvement.

The findings of this updated review have strengthened since the 2005 review by Ernst and Canter [4]. We were also able to locate studies prior to 2005 that were not found by the original SR authors, presumably due to improved database access. As the previous authors reported, the studies are still highly varied and of often questionable quality. There is an ongoing issue of poor reporting, resulting in risks being judged “unclear”; it is unknown whether this hides undeclared risk or is simply an omission of reporting.

This review is not without its own limitations. This review includes all trials aimed at improving health and/or function so we have trials of healthy individuals as well as people with a clinical presentation. We have not included an analysis of publication bias, though we are confident that by using experts in the field and checking grey literature (organizational websites) we have made every effort to capture unpublished (negative) trials. We attempted to account for statistical heterogeneity and can conclude that the analysis for the timed up and go is more robust with the removal of Hillier et al. [23] (Figure 4(b)) because the comparator group differs from the other studies (alternate balance class versus no intervention) and secondly this study was pseudorandomized (allocation based on enrolment day). The question of inactive controls is vexed and permissible when proof of concept or pilot/phase 1 trials are being conducted. We encourage readers to take the stage of research and the design into account in their interpretation.

### 4.2. Implications for Practice

There is promising evidence that FM may be considered for balance classes in ageing populations, both as a preventative approach and for people at risk of falls. There is also some evidence for the use of FM where reduced effort, efficiency of movement, and awareness can play a part in reducing pain or discomfort.

### 4.3. Implications for Research

Further high quality research is required comparing FM to other modalities. Investigations should focus on the impact on self-efficacy, functional independence, and ease and efficiency of functioning, both as strategies for promotion of wellness and wellbeing and also for people with impairment who wish to improve their sense of ease. Mechanisms of effect also need to be investigated. Particular attention needs to be paid to the reporting of best practice trial design and to controlling for a potential placebo effect.

## 5. Conclusions

There is further promising evidence that the FM may be effective for a varied population interested in improving functions such as balance. Careful monitoring of individual impact is required given the varied evidence at a group level and the relatively poor quality of studies to date.

## Figures and Tables

**Table 1:** Example of search strategy.

Number	Searches	Results
1	(Clinical trial or randomised trial or controlled trial).mp. [mp = ab, hw, ti, sh, tn, ot, dm, mf, dv, kw, nm, kf, ps, rs, an, ui]	1900972
2	(Feldenkrais or awareness through movement or functional integration).mp. [mp = ab, hw, ti, sh, tn, ot, dm, mf, dv, kw, nm, kf, ps, rs, an, ui]	2239
3	1 and 2	47
4	Removing duplicates from 3	40

**Table 2:** List of papers excluded with reasons.

Studies	Reason for exclusion
Kirkby (1994)	Controlled trial
Bearman (1999)	Pre/posttest (no control)
Seegert (1999)	Controlled trial
Huntley (2000)	Systematic review
Dunn (2000)	Pre/posttest (no control)
Fialka-Moser (2000)	Commentary
Malmgren-Ohlsen (2001, 2002, 2003)	Controlled trial
Kerr (2002)	Controlled trial
Emerich (2003)	Review
Junker (2003)	Posttest (no control)
Galantino (2003)	Review
Gard (2005)	Review
Mehling (2005)	Review
Liptak (2005)	Review
Batson (2005)	Pre/posttest (no control)
Wennemer (2006)	Pre/posttest (no control)
Porcino (2009)	Descriptive
Mehling (2009)	Review (assessment)
Connors (2010)	Content analysis
Connors (2011a)	Controlled trial
Connors (2011b)	Pre/posttest (no control)
Mehling (2011)	Inquiry (phenomenological)
Ohman (2011)	Pre/posttest (no control)
Laird (2012)	Review
Mehling (2013)	Intervention (not exclusively Feldenkrais)
Gross (2013)	Review
Webb 2013	Pre/posttest (no control)

**Table 3:** Randomised controlled trials of FM (Ernst and Canter, 2005 [4], *n* = 6) with updated RCTs *n* = 14.

Author (year)	Study design	Sample	Intervention	Control	Outcome	Results	Comments
Ruth and Kegerreis (1992) [6]	RCT 2 parallel groups	30 healthy volunteers	Single FM sequence	Participation in other random activities	Degree of neck flexion (goniometer); perceived effort during flexion	Greater degree of neck flexion (goniometer) (*P* < 0.01); less perceived effort during flexion (*P* < 0.05)	Study has pilot character

Johnson et al. (1999) [7]	RCT 2-group crossover (2 phases)	20 people with MS	FM: 8 × 45 min sessions at weekly intervals	8 weeks sham nontherapeutic body work	L and R hand dexterity (pegboard test); 8 symptom/performance scores; 5 mood scales	NSD Less perceived stress following FM (*P* = 0.01)	Positive result could be due to multiple testing for significance

Lundblad et al. (1999) [8]	RCT 3 parallel groups	97 females with neck and shoulder problems	FM: 4 individual sessions, 12 group sessions of 50 mins pw, for 16 weeks, home audio tapes	(C1) physiotherapy 2 × 50 mins per week for 16 weeks; home exercises (C2) no intervention	Clinical assessments (4 measures); physiological tests (18 measures) complaint indices (5 measures); VAS pain ratings (2 measures); disability and sick leave measures (4 measures)	Prevalence of neck pain and disability during leisure decreased in FM versus C1 or C2 (*P* < 0.05) 31 of 33 measures NSD	Important baseline differences, possible regression to the mean. High dropout rate and per protocol analysis. Multiple testing for significance

Stephens et al. (2001) [9]	RCT 2 parallel groups	12 people with MS	FM: 8 × 2–4 hours sessions over 10 weeks	Educational sessions over 10 weeks	3 clinical tests of balance; 3 symptom scales	Significant improvement in FM compared to C for mCTSIB and Balance Confidence Scale; other 4 outcomes NSD	Very small sample size. No baseline data or statistical analysis available

Smith et al. (2001) [10]	RCT 2 parallel groups	26 patients with chronic low back pain	FM: one 30-minute session	Attention control	Pain (McGill); anxiety (STAI)	FM not C reduced affective dimension of pain pre-post (*P* = 0.04) C not FM improved sensory dimension of pain pre/posttest (*P* = 0.03) NSD for evaluative dimension of pain or anxiety	Only acute effects were measured. Baseline differences between FM and C in duration of back pain may be important

Grübel et al. (2003) [11]	RCT 2 parallel groups	66 patients with cancer	FM: 5 × 50 minutes sessions of functional integration in addition to conventional therapies	C: no adjunct therapy	Body image questionnaire; Frankfurter body concept scales; quality of life; sense of movement; and body awareness	Both groups improved in all outcome measures	Nonsignificant trend favoured FM

Additional RCTs							

Brown and Kegerreis (1991) [12]	RCT 2 parallel groups	21 (12 men and 9 women) volunteers pain-free	FM: 45 min audio tape “activating the flexors” lesson	C: listened to the same 45 min audio tape modified to include only instructions pertaining to exercise movements	EMG activity of flexors and extensors (UL) Perception of effort during flexion movement	NSD	There was an overall decrease in mean flexor activity with no change in mean extensor activity for both groups.

Chinn et al. (1994) [13]	RCT 2 parallel groups	23 subjects with upper back, neck, or shoulder discomfort	FM: single ATM lesson; 22 min audio tape	C: single sham treatment; 30 mins gentle neck and shoulder exercises	Functional reach task; perceived effort during the task	NSD Reduced perceived effort in FM group (*P* < 0.05)	Small sample size

Laumer et al. (1997) [14]	RCT 2 parallel groups	30 patients with eating disorder	FM: 9-hour course	C: did not participate in FM	Body Cathexis Scale; Body Parts Satisfaction Scale; Body perception; emotion inventory; Anorexia- Nervosa-Inventory for Self-Rating; eating disorder inventory-2	FM participants showed increasing contentment with regard to problematic zones of their body and their own health and acceptance and familiarity with their body	Full article in German

James et al. (1998) [15]	RCT 3 parallel groups	48 healthy undergraduate students	FM: 4 × 45-minute sessions over 2 weeks of 4 different ATM lessons recorded on audiocassette	Relaxation: 4 × 45 min sessions over 2 weeks listened to relaxation training audiocassette C: no supervised lessons	Hamstring length (modified AKE test)	NSD	Insufficient exposure, low statistical power

Hopper et al. (1999) [16]	Study 1: RCT 2 parallel groups Study 2: subsample of Study 1	Study 1: 75 undergrad physio students Study 2: 39 participants from Study 1	Study 1: FM: single ATM, 45 min audio cassette lesson (no prior FM experience) Study 2: 4 different ATM lessons over 2 weeks	Study 1: C: listened to soft nonverbal music Study 2: same ATM lessons over 4 sessions in 2 weeks when subjects had prior FM experience	Modified AKE test (hamstring length); Sit and Reach test; Borg's 6–20 rating of perceived exertion (during sit and reach test)	Study 1: NSD Study 2: for perceived exertion significant main effect *P* = 0.0003. NSD others	In both studies there was a significant difference in exertion levels between males and females with males exerting more irrespective of group

Kolt and McConville (2000) [17]	RCT 2 parallel groups	54 undergrad physiotherapy students with no prior FM experience	FM: 4 × 45 min ATM lessons via audiocassette over a 2-week period	Relaxation: 4 × 45 min relaxation sessions via audiocassette over a 2-week period C: no specific tasks over 2-week period	Bipolar form of the profile of mood states (POMS-BI)	NSD Composed-anxious scores of the POMS-BI did vary significantly over time (*P* = 0.001) for all participants. Females in FM and relaxation groups reported significantly lower anxiety scores at completion compared with control	No differences between FM and relaxation groups

Löwe et al. (2002) [18]	Pseudorandomized, consecutive allocation	60 patients transferred to normal ward after acute treatment for MI	FM: 2 × 30 min individual sessions	Relaxation: 2 × 30 min individual PMR C: no body-oriented interventions	Body image questionnaire (FKB-20, German version); Hospital Anxiety and Depression Scale-German version (HADS-D); Munich Quality of Life Dimensions List (MLDL); German version Generalized Self-Efficacy Scale (GSES)	NSD	Overall improvements were seen in MLDL, GSES, and FKB-20

Stephens et al. (2006) [19]	RCT 2 parallel groups	38 graduate students	FM: 5 × 15 min ATM sessions/wk, audiotape over 3-week period	C: regular daily activities	AKE (hamstring muscle length)	Significant increase in hamstring muscle length (*P* = 0.005) in ATM group compared with control	Participants varied greatly in the duration and number of home sessions completed

Quintero et al. (2009) [20]	RCT 2 group (crossover design for control)	3- to 6-year-old children with sleep bruxism	FM: 3 hr sessions × 10 during 10-week period based on ATM	C: no details	Various measures of joint function; nocturnal bruxism	Statistically significant increase of CVA angle (*P* = 0.0) for FM c.f. C. After intervention 77% parents in FM reported no nocturnal bruxism c.f. 15.38% for C	At baseline two groups were comparable

Vrantsidis et al. (2009) [21]	RCT 2 groups (crossover design for control)	55 participants aged ≥55 years	FM: getting grounded gracefully program (based on ATM) 2 × 40–60 min sessions/wk over 8 weeks	C: continue with usual activity	Frenchay Activity Index; Human Activity Profile; Assessment of Quality of Life; Modified Falls Efficacy Scale; Abbreviated Mental Test Score; four-square step test; timed up and go test; the Step Test; Timed Sit-To-Stand Test; Clinical Stride Analyzer; force-platform measures of gait, mobility, and function; satisfaction survey	Significant effects for gait speed (*P* = 0.028) and Modified Falls Efficacy Scale (*P* = 0.003) for FM group; near significant effect for timed up and go test (*P* = 0.056) Positive feedback from survey	No significant baseline differences between groups. High class attendance

Ullmann et al. (2010) [22]	RCT 2 groups	47 relatively healthy independently living ≥65-year-olds	FM: 1 hour ATM sessions 3x/week for 5 weeks (provided by instructor)	C: waitlist	Falls Efficacy Scale; Activities Specific Balance Confidence Scale; timed up and go and TUG with added cognitive task; GAITRite Walkway System; tandem stance	Balance (*P* = 0.030) and mobility (*P* = 0.042) increased for FM, whilst fear of falling decreased (*P* = 0.042).	At baseline groups comparable except for higher BMI in intervention group

Hillier et al. (2010) [23]	Pseudorandomized control trial 2 groups	22 healthy people postretirement	FM: ATM class, 1 hr/week for 8 weeks	C: generic balance class 1 hr/week for 8 weeks	SF-36; Patient Specific Functional Scale (PSFS); timed up and go test; functional reach test (FRT); Single Leg Stance Time (SLS); Walk on Floor Eyes closed (WOFEC)	Significant time effect for all measures except for WOFEC Significant improvements for both groups for SF-36, PSFS, and FRT. SLS improved FM (*P* = 0.016)	Post hoc individual analysis comparisons made

Bitter et al. (2011) [24]	RCT 3 arms	29 healthy university students	FM1: ATM lesson 1 × 40 min, dominant hand; FM2: same but nondominant hand	C: relaxation lesson 1 × 40 min	Purdue Pegboard Test; Grip-lift test; subjective changes	FM1 significant group by time intervention effect when compared to control group for dexterity	

Nambi et al. (2014) [25]	RCT 3 arms	60 institutionalized ageing people	FM: ATM classes 3 × 6 weeks	PI: Pilates classes 3 × 6 weeks C: sham walking 3 × 6 weeks.	Functional reach test; timed up and go test; Dynamic gait index; RAND-36 for quality of life	Both FM and PI improved all measures (*P* < 0.000); C improved TUG and DGI only	

RCT: randomised controlled trial; FM: Feldenkrais Method; MS: multiple sclerosis; L: left; R: right; C: control; pw: per week; VAS: visual analogue scale; mCTSIB: Modified Clinical Test of Sensory Integration and Balance; NSD: no significant difference; STAI: State/Trait Anxiety Index; EMG: electromyography; UL: upper limb; ATM: awareness through movement (lesson); min: minutes; AKE: active knee extension test; MI: myocardial infarct; PMR: progressive muscle relaxation; c.f.: compared with; SF-36: short form 36; PI: Pilates.
